# Analysis of a Flexible Photoconductor, Manufactured with Organic Semiconductor Films

**DOI:** 10.3390/mi15040446

**Published:** 2024-03-27

**Authors:** Luis Alberto Cantera Cantera, María Elena Sánchez Vergara, Leon Hamui, Isidro Mejía Prado, Alejandro Flores Huerta, Teresa Lizet Martínez Plata

**Affiliations:** 1 Faculty of Engineering, Universidad Anáhuac México, Av. Universidad Anáhuac 46, Col. Lomas Anáhuac, Huixquilucan 52786, Mexico; lcanterac@ipn.mx (L.A.C.C.); leon.hamui@anahuac.mx (L.H.);; 2 Instituto Politécnico Nacional—ESIME, Unidad Profesional Adolfo López Mateos, Av. Luis Enrique Erro S/N, Gustavo A. Madero, Zacatenco 07738, Mexico; 3 Universidad Politécnica de Cuautitlán Izcalli, Av. Lago de Guadalupe, Colonia Lomas de San Francisco Tepojaco, Cuautitlán Izcalli 54720, Mexicolizetplata@gmail.com (T.L.M.P.)

**Keywords:** organic semiconductor, organic photoconductor, planar heterojunction, flexible device, electrical properties

## Abstract

This work presents the evaluation of the electrical behavior of a flexible photoconductor with a planar heterojunction architecture made up of organic semiconductor films deposited by high vacuum evaporation. The heterojunction was characterized in its morphology and mechanical properties by scanning electron microscopy and atomic force microscopy. The electrical characterization was carried out through the approximations of ohmic and SCLC (Space-Charge Limited Current) behaviors using experimental J–V (current density–voltage) curves at different voltages and under different light conditions. The optimization of the photoconductor was carried out through annealing and accelerated lighting processes. With these treatments, the Knoop Hardness of the flexible photoconductor has reached a value of 8 with a tensile strength of 5.7 MPa. The ohmic and SCLC approximations demonstrate that the unannealed device has an ohmic behavior, whereas the annealed device has an SCLC behavior, and after the optimization process, an ohmic behavior and a maximum current density of 0.34 mA/mm^2^ were obtained under blue light. The approximations of the device’s electron mobility ($\mu_{n}$) and free carrier density ($n_{0}$) were performed under different light conditions, and the electrical activation energy and electrical gap were obtained for the flexible organic device, resulting in appropriate properties for these applications.

## 1. Introduction

One of the main motivations in Organic Electronics development is the potential to fabricate optoelectronic devices at low cost, with less use of polluting processes and at lower temperatures, compared with silicon-based technologies. Silicon, for example, needs to be purified by costly procedures in a controlled inert atmosphere to obtain the rigid wafers. However, organic semiconductors (OSs), including polymers [1,2] and small molecules [1,3], can be thermally evaporated in vacuum methodologies at a lower cost to generate high-purity thin films [4,5,6]. These processes do not require high temperatures, and the OS can be processed on rigid or flexible substrates, opening the door toward flexible electronics. Among the main characteristics of flexible or conformable electronics, the manufacture of OS thin films on flexible and transparent non-flat surfaces has been studied [1,7,8,9,10]. OS is mainly composed of polymers or small molecules that have semiconductor properties, such as silicon-based materials. However, the OS owes its electrical conductivity to the electronic delocalization along each molecule π-conjugated structure, which interacts with others along the semiconductor film. The interaction occurs mainly through van der Waals forces and Coulombic interactions due to small dipole and/or quadrupole moments within the molecules. The electronic coupling between molecules is a very important parameter in the charge carriers’ mobility in the OS and is related to the strength of the interactions of neighboring molecules [11].

For the application of OS in electronics and, specifically, in organic optoelectronic devices, two of the processes of greatest interest are the charge injection from the device work electrode to the OS and the mobility of the charge carriers. The efficiency of the injection process depends on the type of electronic device. Among the simplest optoelectronic devices are the photoconductors, whose main function is the variation of their electrical conductivity with the incident light [12]. Its operation is summarized in different stages: surface electrostatic charging, charge carrier photogeneration, and transport along the semiconductor films that comprise it. In terms of the semiconductor material, it must be a good photo-generator of electrical charges and fast enough so that the photogenerated charge carriers pass through the device in a short time. When the semiconductor absorbs light, the number of free electrons and holes must increase, resulting in greater electrical conductivity. The above is directly related to the mobility of charge carriers, a very important physical parameter, which implies the speed per unit of the electric field. It is important to consider that, to cause the required excitation of charges, the light striking the semiconductor must have sufficient energy to excite the electrons across their HOMO (highest occupied molecular orbital)-LUMO (lowest unoccupied molecular orbital) energy gap or to excite impurities within this energy gap.

The study of conductivity and charge carrier mobility on organic semiconductors is fundamental for defining the electrical properties of electronic devices. In the literature, various charge transport behaviors and models have been reported, as well as different methods for characterizing them [13,14,15,16]. In this work, the charge carrier’s mobility is studied through ohmic and space-charge-limited current (SCLC) definitions, which describe the behavior of the I-V curves [12,17,18]. The ohmic regime or linear region is generally produced at low voltages, and it is modeled by:

(1) $$J_{\Omega }=qn_{0}\mu_{n}\frac{V}{d_{s}}$$

whereas the SCLC regimen is quadratic and is defined by:

(2) $$J_{SCLC}=\frac{9}{8}\mu_{n}\varepsilon_{0}\varepsilon_{r}\frac{V^{2}}{d_{s}^{3}}$$

where J denotes the current density, q is the electronic charge, $n_{0}$ is the free carrier density, $\mu_{n}$ is the electron mobility, V is the applied voltage, $d_{s}$ is the thickness of the sample, $\varepsilon_{0}$ is the vacuum permittivity and $\varepsilon_{r}$ is the dielectric constant. Equations (1) and (2) describe the behavior of the current density through the device, and it is important to consider that Equation (2) exhibits a growth rate greater than Equation (1), which means that the current density in the SCLC regimen growth faster than the ohmic regimen.

Many variants of photoconductive materials are currently being investigated to improve their quality, functions, properties, processing, generation, and electrical charge transport. Initially, inorganic photoconductors made of amorphous selenium and hydrogenated amorphous silicon were used. For economic, flexibility, functional, and low environmental impact reasons, the current trend involves the use of photoconductive materials such as OS. These semiconductors’ properties can be chemically modified relatively easily by combining different types of organic molecules. Also, their behavior can be enhanced directly in photoconductive devices with the use of hole-transporting (HTL) and electron-transporting (ETL) interfacial layers. It is due to the above that organic optoelectronic devices are manufactured, and study must be promoted to complement the manufactured silicon-based devices in different applications. The objective of this work is to manufacture an organic photoconductive device (OPD) composed of various layers, where copper phthalocyanine (CuPc) and bathocuproine (BCP) were used as hole and electron transport layers, respectively. The CuPc is a popular hole transport material, which usually serves as a donor and absorber material [19]. The BCP possesses high electron mobility and a wide band gap [19,20,21] and acts as the ETL to separate the cathode and the electron acceptor (EA) film in the device. The BCP improves electron transport because of its energetic interactions with the cathode [21,22,23]. Regarding the electrodes, they directly influence the built-in electrical field and the electronic parameters like charge carriers’ mobility of the photoconductor device due to the work function (φ) difference between them [19,24,25]. In this study, indium tin oxide (ITO) has been used as an anode due to its transparency and high transmittance in the visible range [8,19,26,27]. Ag has been used as a cathode due to its common use as a counter electrode for ITO in organic devices [1,19,28] and its relatively close φ (4.8 and 4.2 eV for ITO and Ag, respectively). Moreover, in the device proposed in this study, chloro indium(III) phthalocyanine (InClPc) was proposed as the AE because it has been very little studied as a precursor for OPD [15]. The diamagnetic indium(III) metal cation was selected as the central metal in the phthalocyanine cavity to enhance the photophysical and electrical properties [29,30,31]. Finally, the MEH-PPV was used as an electron donor (ED), and it is important to mention that the device was manufactured on a flexible polyethylene terephthalate (PET) substrate. Among flexible substrates, PET is usually a good candidate due to its low coefficient of thermal expansion, its chemical resistance, and its low cost, among other characteristics that facilitate the manufacture of OPD [1,32,33,34,35,36]. It is for this reason that PET was used in the device manufacture in this work with a planar heterojunction architecture. The study includes the evaluation of the photoconductor response of the device under different lighting and temperature conditions. Furthermore, to optimize its electrical performance, the device was annealed and subsequently subjected to accelerated lighting conditions.

## 2. Materials and Methods

**Figure 1:**
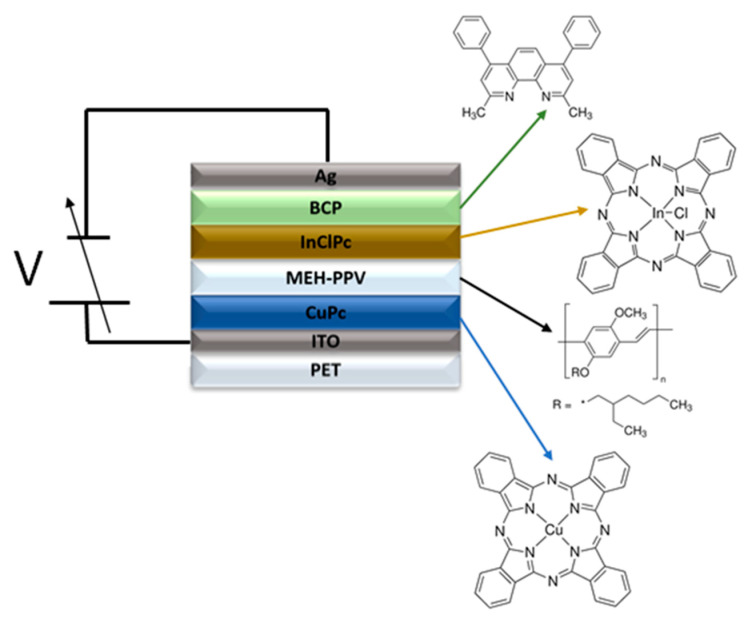
Device structure.

CuPc (Copper(II) phthalocyanine: C_32_H_16_CuN_8_), MEH-PPV (poly[2-methoxy-5-(2′-ethylhexyloxy)-1,4-phenylene vinylene: (C_18_H_28_O_2_)_n_), InClPc (chloride indium(III) phthalocyanine: C_32_H_16_ClInN_8_) and BCP (bathocuproine: C_26_H_20_N_2_), were obtained directly from commercial sources (Sigma-Aldrich, Saint Louis, MO, USA), and did not require purification prior to use. Subsequently, the planar heterojunction device shown in Figure 1 was manufactured, and for this device, a commercial polyethylene terephthalate (PET) substrate coated with indium tin oxide film (ITO: In_2_O_3_/(SnO_2_)_x_) that acted as an anode was used. As mentioned earlier, the CuPc acted as the HTL, the MEH-PPV served as the ED, the InClPc as the EA, the BCP as the ETL, and the Ag as the cathode. The films were deposited using a high vacuum thermal sublimation system with two evaporation ports, in which molybdenum crucibles with different compounds were installed. The use of vacuum sublimation provides a high degree of variable control during deposition, such as time, sublimation temperature, substrate temperature, and base pressure. Optimization of these variables allows the film’s deposition with precise thickness and molecular orientation [37]. For the CuPc, an 8.6 × 10^−6^ Torr vacuum was used. For the MEH-PPV polymer, a 5 × 10^−6^ Torr vacuum was used. For the InClPc, an 8.6 × 10^−5^ Torr vacuum was used, and for the BCP, a 9 × 10^−5^ Torr vacuum was used, obtaining a 143 Å, 32 Å, 15 Å, and 2 Å film thickness, respectively. Thicknesses were measured with a quartz crystal microbalance monitor connected to a thickness sensor. It should be noted that after the device manufacture and before the cathode deposit, the device was annealed in a Briteg SC-92898 (Instrumentos Científicos, S.A de C.V. México City, México) oven at 200 °C for 30 min. This annealing was made to carry out a recrystallization process in the films because they were manufactured by the vacuum thermal sublimation technique that generates thermal shock conditions of the molecules in the gaseous state with the substrate that tends to lose their arrangement, obtaining an amorphous structure [37]. The topography, roughness, and some mechanical parameters of the planar heterojunction structure have been studied with an atomic force microscope (AFM) using an Ntegra platform (Nanosurf AG, Liesta, Switzerland). Subsequently, the images were analyzed using Gwyddion 2.65 software. To carry out the surface morphological characterization of planar heterojunction, a ZEISS EVO LS 10 (Carl Zeiss AG., Jena, Germany) scanning electron microscope (SEM) operated at a voltage of 20 kV and a focal distance of 25 mm was used. The electrical properties in the heterojunction device were carried out by the four-probe method using a sensor station with a Next Robotix lighting controller circuit (Comercializadora K Mox, S.A. de C.V., Mexico City, Mexico) and a Keithley 4200-SCS-PK1 auto-ranging picoammeter (Tektronix Inc., Beaverton, OR, USA). This is a technique used commonly for resistivity measurements, which is performed along a line over the material with equal spaces between the test points. The device was irradiated with an illumination system of light-emitting diodes, which allowed irradiation with seven different types: UV (2.70 eV), blue (2.64 eV), white (2.57 eV), green (2.34 eV), yellow (2.14 eV), orange (2.0 eV) and red (1.77 eV). Additionally, the electrical properties were measured under the influence of temperature with a Next Robotix sensing station from 20 °C to 150 °C and with measurement intervals of 10 °C. Finally, the device was subjected to accelerated lighting conditions to provide energy to the molecules HOMO electrons that form the different layers. These conditions were carried out for 3 h with a Dukane 28A653A Bias lamp of 360 watts and 82 V.

## 3. Results and Discussion

### 3.1. Morphological Characterization of Heterostructure

**Figure 2:**
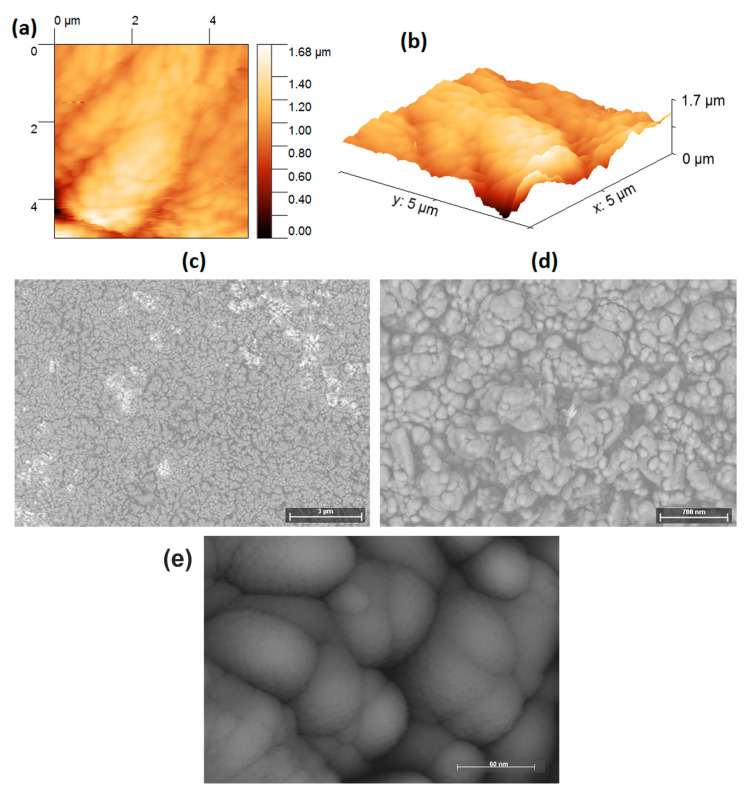
(**a**) 2D and (**b**) 3D AFM images at 5 × 5 µm. SEM images at (**c**) 250×, (**d**) 1000×, and (**e**) 3000× of planar heterojunction.

AFM measurement in tapping mode was carried out to determine the topography of the planar heterojunction fabricated with the four films, CuPc, MEH-PPV, InClPc, and BCP. Figure 2a,b shows the 2D and 3D images, respectively, of the heterojunction surface and segregated rounded structures were observed. Table 1 shows the Average roughness (Ra) and the Root Mean Square roughness (RMS), where their values are high due to the presence of the four films that form the heterojunction structure but mainly due to the morphological conditions of its last BCP layer. Figure 2b shows a depth variation of approximately 1.7 μm from the valley to the highest point of the heterojunction surface. However, a uniform surface with 437 nm Ra is observed. To complement the above and to obtain more information about the heterojunction morphology, SEM was carried out and is shown in Figure 2c–e at 250×, 1000×, and 3000×, respectively. According to SEM images, the surface of the heterojunction has a morphology made up of rounded structures that extend continuously on the film and tend to form clusters or aggregates. The clusters vary in size, and most of them are smaller than 2 μm, where rounded structures larger than 100 nm are observed. However, the rounded structures (Figure 2d) vary in size and the way they attach, generating an apparent depth variation in the surface morphology, similar to what is observed in AFM images (Figure 2a,b). It is important to note that there is adequate connectivity between the clusters, which can favor the transport of electric charges among them and, therefore, on the film. Figure 2e shows a micrograph with higher magnification and resolution, where a more detailed morphology of the rounded structures and their attachment is observed. Very small and rounded structures (~15–20 nm) with an almost smooth surface were observed, but others have grown into larger sizes. The clusters present continuity to adjacent and piled clusters, which supports the previous hypothesis. The large size of the grains combined with the good continuity of the films is a good approach to improve charge transport and mobility. The surface conditions of the planar heterojunction are important because, when manufacturing the optoelectronic device, it is the last BCP layer that will be in contact with the silver cathode through which the electrical charges will be injected.

**Figure 3:**
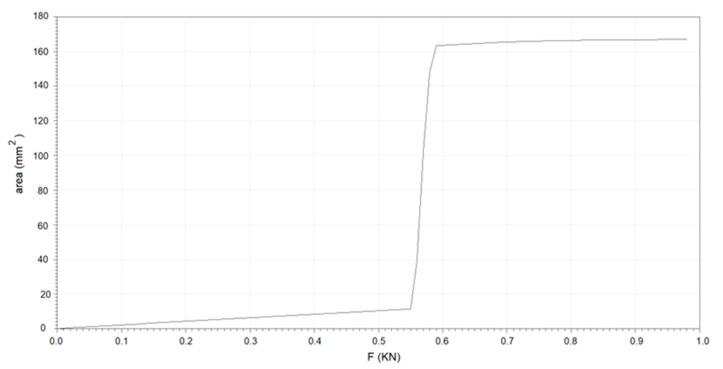
Relationship between the adhesion force and the area.

Additionally, to obtain some mechanical parameters of the planar heterojunction, Force Spectroscopy was used, which is a method used in Nanosurf AFMs and refers to a measurement in which the cantilever approaches and indents the planar heterojunction surface and then withdraws. During this measurement, the cantilever deflection vs. piezo movement is measured, and this can be converted to a force vs. tip-sample separation measurement that provides mechanical information about the planar heterojunction. AFM force curves can be used for various mechanical parameters extraction for the planar heterojunction, including the relationship of adhesion force (F), mechanical stress (*σ*), stretching or deformation (*ε*), and indentation depth for Knoop Hardness (HK). The relationship between F and area is presented in Figure 3. It is interesting to mention that an F of around 0.55 N is maintained for practically all areas and that a maximum F of around 0.99 N occurs in the largest area. On the other hand, it is observed in Table 1 that although the maximum *σ* is high, which translates into low *ε*, the HK of the heterojunction is very low. This may result in premature fatigue of the planar heterojunction under service conditions. Due to the above, the heterojunction was annealed, and according to Table 1, its hardness increased significantly. Although the *ε* increases and the *σ* decreases, their values remain in the same order of magnitude, and the increase in hardness favors the resistance of the heterojunction under service conditions.

### 3.2. Electrical Characterization of the Device

**Figure 4:**
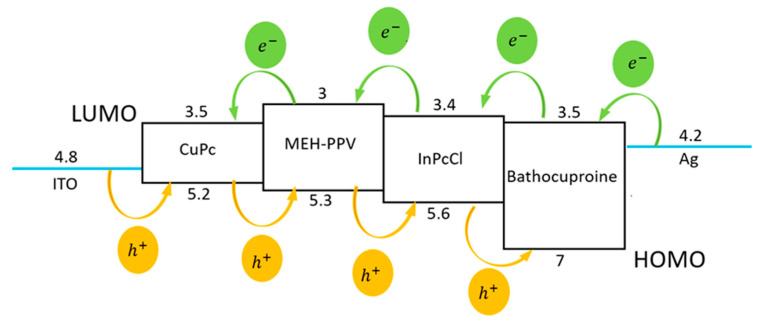
Schematic representation of the relative distribution of Highest Occupied Molecular Orbital (HOMO) and Lowest Unoccupied Molecular Orbital (LUMO) energetic levels of the device components.

The flexible photoconductive device was manufactured on PET according to the Figure 1 scheme. The injection and transport of holes and electrons in the device could be explained as the charge carriers jumping from one layer to a neighboring layer [25] (see the energy levels in Figure 4). The ITO acted as an anode, with Fermi level and work function near the HOMO orbital energy of CuPc (5.2 eV). The energy barrier between the ITO and CuPc is 0.4 eV. The hole transport layer was applied to lower the energy barrier between the ITO and the MEH-PPV electronic donor. On the other hand, the CuPc HOMO is almost aligned with the 5.3 eV HOMO of MEH-PPV. The InClPc acceptor has a 5.6 eV HOMO, which is very close to that of MEH-PPV (energy barrier of 0.3 eV) and higher than the 7 eV of BCP. Finally, Ag was deposited over the device, where its Φ_f_ = 4.2 eV is close to that of ITO and would provide a field that promotes hole collection at the ITO and electron collection at the Ag. According to Figure 4, the cathode extracts the electrons coming from the BCP. This molecule facilitates the electron transport that the InPcCl attracts from donor MEH-PPV. The HOMO of MEH-PPV and the HOMO of InPcCl present a suitable energy correspondence, which allows the phthalocyanines to favor the hole transport injected by the anode.

**Figure 5:**
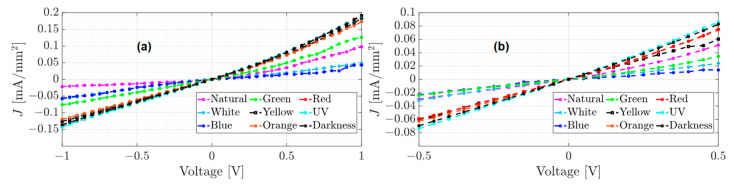
J–V curves of the unannealed film under different lighting conditions (**a**) test from −1 to 1 volt and (**b**) test from −0.5 to 0.5 volts.

**Figure 6:**
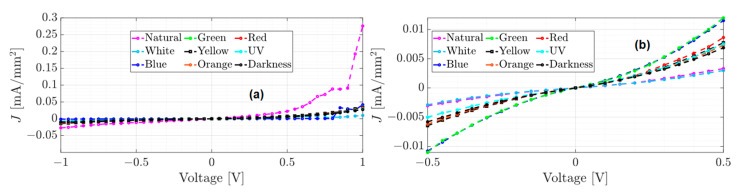
J–V curves of the annealed film under different lighting conditions (**a**) test from −1 to 1 volt and (**b**) test from −0.5 to 0.5 volts.

**Figure 7:**
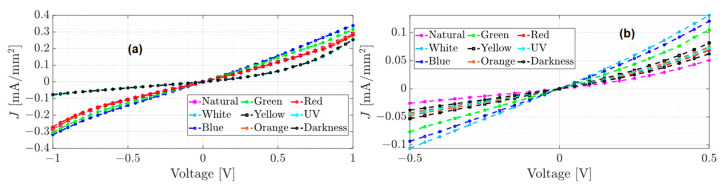
J–V curves of the annealed film subjected to accelerated lighting conditions under different lighting conditions (**a**) test from −1 to 1 volt and (**b**) test from −0.5 to 0.5 volts.

The unannealed, annealed, and annealed subjected to accelerated lighting conditions devices were electrically characterized and tested from −1 to 1 volt and from −0.5 to 0.5 volts under different electromagnetic radiation: darkness, natural and artificial white light conditions. Red, orange, yellow, green, blue, and UV light were also measured to determine the individual effect of each of the radiations that constitute the UV-vis spectrum. Figure 5, Figure 6 and Figure 7 show the J–V curves of the unannealed, annealed, and subjected to accelerated lighting conditions devices under different lighting conditions, respectively.

Comparing the order of magnitude of the current density achieved in the J–V curves for each device, it is evident that each manufacturing process induces changes in the conduction current. For instance, the unannealed device reached a maximum current density of 0.19 mA/mm^2^ under the red light and a minimum current density of 0.043 mA/mm^2^ under blue light (see Figure 5a). After an annealing process, the current density decreased significantly compared to an unannealed device (see Figure 5 and Figure 6). However, there was an increase under natural light conditions, where a current density of 0.28 mA/mm^2^ was obtained. According to Table 1, this may be due to the increase in the roughness and hardness of the heterojunction since, on the one hand, the high roughness can generate a dissipation of electrical charges and, on the other hand, the greater rigidity between the molecules that integrate the layers could generate a lower flow of charges. However, when the annealed device was subjected to accelerated illumination conditions, a maximum current density of 0.34 mA/mm^2^ was obtained under blue light and a minimum of 0.25 mA/mm^2^ in darkness (see Figure 6a). This is due to the electronic excitation generated by the high radiation, which facilitates the transport of charges through the films that make up the device.

On the other hand, all devices manufactured present photoconductor properties because the current density present changes under different lighting conditions [12]. It is important to emphasize that the shape of the J–V curves changes for each manufacturing process. The J–V curve of the unannealed device (see Figure 5) exhibits ohmic or linear behavior, whereas the J–V curve of the annealed device has a steeper growth rate with a SCLC behavior (see Figure 6). In the case of the annealed device subjected to accelerated illumination conditions, some curves display ohmic behavior while others show SCLC behavior (see Figure 7). To approximate the electron mobility $\mu_{n}$ and the free carrier density $n_{0}$, the least squares fitting of Equations (1) and (2) for ohmic and SCLC behaviors, respectively, is presented below.

Since Equation (1) for ohmic behavior represents a straight line without an independent coefficient, its slope can be defined as follows:

(3) $$a_{1}=\frac{qn_{0}\mu_{n}}{d_{s}}\;$$

On the other hand, as Equation (2) represents a parabola centered at the origin, the only parameter to estimate is determined by:

(4) $$a_{2}=\frac{9\mu_{n}\varepsilon_{0}\varepsilon_{r}}{8d_{s}^{3}}$$

To assess the quality of the ohmic and SCLC models’ approximation to the experimental data, the Root Mean Square Error (RMSE) will be used, as defined by

(5) $$\mathrm{RMSE}=\sqrt{\frac{1}{n}\sum_{i=1}^{n}{\left(c_{i}-m_{i}\right)}^{2}}\;$$

where $c_{i}$ is ith calculated value, and $m_{i}$ is the ith measured value. It is important to emphasize that both approximations, ohmic and SCLC, were attempted, but only the one with the lower approximation error was reported.

After estimating the parameters, $a_{1}$ and $a_{2}$, the electron mobility and free carrier density can be calculated using the following equations.

(6) $$\mu_{n}=\frac{8d_{s}^{3}a_{2}}{9\varepsilon_{0}\varepsilon_{r}}$$

(7) $$n_{0}=\frac{a_{1}d_{s}}{q\mu_{n}}$$

Using the experimental data from Figure 5, Table 2 presents the approximation of the parameter $a_{1}$ for ohmic behavior of the unannealed device for tests 1 and 2, ranging from −1 to 1 V and from −0.5 to 0.5 V, respectively, under different light conditions, as well as the RMSE index for each approximation.

**Figure 8:**
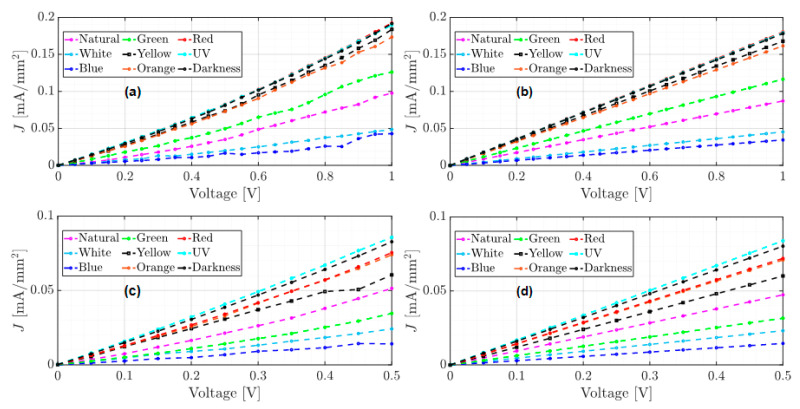
J–V curves of the ohmic behavior approximation of the unannealed device, (**a**,**c**) measured data, and (**b**,**d**) approximated data.

From Table 2, it can be observed that the approximation of the slopes is similar in both tests and for each type of light used. This implies that the growth rate in both tests is also similar. Using the estimates from Table 2, the graphs of the approximation results for the unannealed device’s tests 1 and 2 are shown in Figure 8. In the ohmic regime fitting of Test 1, from 0 to 1 V, the experimental data are shown in Figure 8a, and the calculated data are displayed in Figure 8b. For Test 2, from 0 to 0.5 V, the experimental and calculated data are shown in Figure 8c,d, respectively.

The approximations of the J–V curves shown in Figure 8b calculate maximum and minimum current densities of 0.18 mA/mm^2^ and 0.034 mA/mm^2^ under red and blue light, respectively, which have a difference of 0.01 compared to the measured data. However, the estimates from Test 2 in Table 2 have a lower RMSE in comparison to Test 1 and were used for free carrier density calculation.

**Figure 9:**
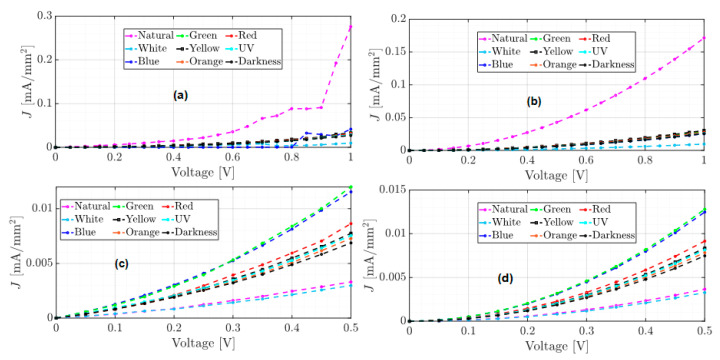
J–V curves of the SCLC behavior approximation of the annealed device, (**a**,**c**) measured data, and (**b**,**d**) approximated data.

On the other hand, as the J–V curve of the annealed device (see Figure 6) exhibits a higher growth rate than that of the unannealed device, it was approximated using Equation (2) for SCLC behavior. Table 3 shows the results of the estimations and the RMSE errors for tests 1 and 2. Figure 9 shows the J–V approximation curves for each test.

As mentioned above, the experimental results of the annealed device show a significant decrease in the conduction current compared to the unannealed device. However, since the SCLC behavior model best fits the experimental data (see Figure 9), the order of the current conduction growth rate has increased. On the other hand, results from Table 2 indicate that the best approximation of the SCLC behavior was achieved with data from Test 2, as the RMSE value was lower compared to Test 1. Therefore, these estimates will be used to determine electron mobility.

**Figure 10:**
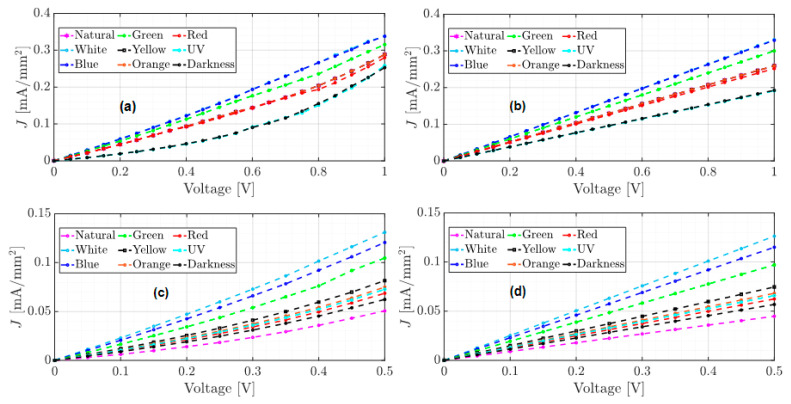
J–V curves of the ohmic behavior approximation of the annealed device subjected to accelerated illumination conditions, (**a**,**c**) measured data, and (**b**,**d**) approximated data.

To enhance the conduction of the annealed device, it was subjected to accelerated illumination conditions, and as shown in Figure 7, the current density reached a maximum of 0.34 mA/mm^2^ when the device was exposed to blue light. Although there was an increase in current density compared to the other two devices, in Figure 7a, it can be observed that the J–V curves exhibit ohmic behavior, except for those exposed to UV light and darkness, which show SCLC behavior. The estimates of the ohmic behavior approximation to the experimental data for the annealed device subjected to accelerated illumination conditions are presented in Table 4. The approximation curves obtained are shown in Figure 10.

Although the J–V curves in Figure 10a,c show an apparent SCLC behavior, the RMSE of the ohmic model was lower in all cases, except for the device under UV light and darkness from Figure 10a. On the other hand, comparing the RMSE errors from Table 4, the best fit of the ohmic model was achieved with the experimental data from Test 2.

After obtaining the estimates of parameters $a_{1}$ and $a_{2}$ under different light conditions, the electron mobility $\mu_{n}$ and free carrier density $n_{0}$ can be easily calculated using Equations (6) and (7), respectively. Using estimates of Test 2 from Table 3 and considering that $q=1.6\times 10^{-19}\;C$, $\varepsilon_{r}=5,$ $\varepsilon_{0}=8.85418\times 10^{-12}\;\frac{C}{Vm}$ and $d_{s}=1.92\times 10^{-8}\;m$, Table 5 shows the resulting electron mobility in $\frac{mm^{2}}{Vs}$.

Electron mobility is one of the most important parameters for analyzing and characterizing semiconductors, whether organic or inorganic, as it is crucial for device conductivity. In general, electron mobility in organic semiconductors is lower than in inorganic ones. However, if an organic semiconductor exhibits a mobility between 0.1 and 1 $\frac{{cm}^{2}}{Vs}$, it is a good semiconductor device [38]. The results of the electron mobility calculation for the manufactured device, as shown in Table 5, exhibit an order of magnitude of $10^{-5}\;\frac{{cm}^{2}}{Vs}$, which falls within the range of values reported in various studies on the characterization of organic semiconductors, ranging from the order of magnitude of $10^{-7}$ to 1.66 $\frac{{cm}^{2}}{Vs}$ [38,39,40].

Just like electron mobility, the free carrier density of a semiconductor is also a crucial parameter for the device’s conductivity. Based on several reports, it has been found for organic semiconductors that the carrier density order of magnitude can vary between $10^{18}$ and $10^{21}$ $\frac{1}{{cm}^{3}}$ [41]. From Table 6, the manufactured device has a free carrier density of the order of $10^{18}$ $\frac{1}{{cm}^{3}}$. This indicates that it falls within the range reported for other organic semiconductors.

**Figure 11:**
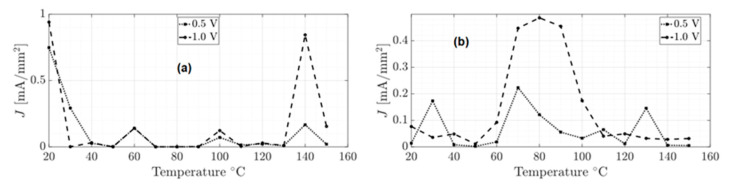
Current density of the unannealed (**a**) and annealed (**b**) devices subjected to different temperature values.

On the other hand, the unannealed and annealed devices were exposed to different temperatures to explore their current density conduction. They were exposed to temperatures from 20 to 150 °C and supplied with 0.5 V and 1.0 V. Figure 11 shows the current density behavior to different temperature values.

From Figure 11, the unannealed device exhibits a low current density value for temperatures ranging from 60 to 90 °C. However, after the annealing process, the current density of the device increases for temperatures ranging from 70 to 90 °C. This means that the annealing process improved the device’s conduction at high temperatures. Specifically, when the device was powered with 1 V, a maximum current density of 0.49 mA/mm^2^ was reached at 80 °C, while when powered with 0.5 V, the device reached a maximum current of 0.22 mA/mm^2^ at 70 °C.

**Figure 12:**
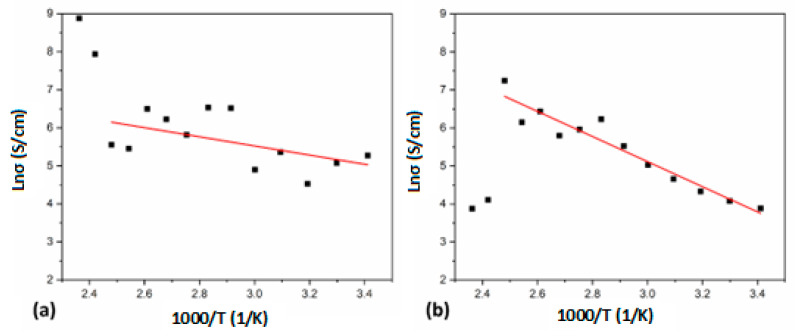
Electric conductivity as a function of temperature for the photocurrent device (**a**) unannealed and (**b**) annealed.

Finally, in the CuPc/MEH-PPV/InClPc/BCP device, electrical conductivities (*σ*) were measured in the 290–423 K temperature range. The obtained *σ* were around 10–10^2^ S/cm. These resulting values are within the semiconductor region (10^2^ to 10^3^ S/cm) [42,43]. These results consider uniform film homogeneity through the conduction channel length. To obtain additional information related to the device’s conduction mechanisms, the conductivity as a function of the temperature was plotted and shown in Figure 12. The conductivity has the general form,

(8) $$\sigma =\sigma_{0}\exp\left(-\frac{E_{a}}{kT}\right)$$

where *σ*_0_ is the pre-exponential factor, *E_a_* is the thermal activation energy of the electrical conductivity, *T* is the absolute temperature, and *k* is Boltzmann’s constant (1.38 × 10^−23^ J/K). Figure 10a shows a plot of ln(*σ*) against the inverse of the temperature, where data points were linearly fitted to calculate the thermal activation energy of the device’s electrical conductivity without thermal treatment [44,45]. The temperature range used allows intrinsic region and extrinsic region conduction mechanisms. Depending on the temperature region, the charge conduction mechanism is considered to be free band type and hopping. First, it is observed that the conductivity increases with the temperature. Thus, the number of carriers and mobility increases. Then, for the highest temperatures (>135), the data points show a step variation in the conductivity (Figure 12a), indicating that either the device may have changed the conduction mechanism or the device interphases and films were affected by the temperature. For the first case, a change from a charge carrier transport in localized states within the bandgap to a charge carrier transport in extended states, moreover, as a change from extrinsic to intrinsic conduction. On the other hand, a rearrangement in the film or crystallization may change the conduction processes, whereas the interface quality also changes the device conductivity. However, by doing the thermal treatment, resulting *σ* values of approximately 10 to 10^3^ S/cm were obtained. Also, for higher temperatures, a step variation to lower values was observed that could be related to a damaged device, especially related to the PET substrate. This is a drawback of flexible electronics, as the poor resistance of the substrates to high temperatures affects the device’s performance.

An activation energy of 0.12 eV for the unannealed device and of 0.28 eV for the annealed device was calculated from Figure 12a,b slopes. These results are in accordance with other similar devices conformed mainly by CuPc [44,46]. The latter indicates that the thermal activation energy *E_a_* is increased by thermal annealing (crystallization process). The crystallization process reduces the defect state density within the gap, resulting in a higher activation energy, but the conductivity might also be reduced. By comparing the results shown in Figure 10a,b, a reduction in the conductivity was observed for low temperatures. In the intrinsic region, the value of the thermal, electrical bandgap can be calculated as double the activation energy [45], so it resulted in 2.29 eV for the device without treatment, which is close to the reported optical bandgap for the CuPc [46,47]. Thus, the presented device shows appropriate electronic properties for flexible photoconductive applications.

## 4. Conclusions

An organic photoconductor with dispersed heterojunction architecture was manufactured, and it was annealed and irradiated under accelerated illumination conditions to increase the current transported in it. The electrical tests under different light conditions demonstrated that the manufactured device has photoconductor properties because the current density changed when it was exposed to different types of light. On the other hand, concerning the J–V curves of the unannealed device, a maximum current density of 0.19 mA/mm^2^ was achieved under red light, and as the experimental data exhibit a linear behavior, they were approximated using the equation for ohmic behavior. After an annealing process, the current density growth rate of the device increased because the SCLC behavior equation best fitted the J–V curves of the device; however, there was a decrease in the current conduction of the device. Despite the decrease in current conduction, with this approach and Equation (6), the electron mobility $\mu_{n}$ of the device could be determined under different lighting conditions, which is on the order of $10^{-5}\;\frac{{cm}^{2}}{Vs}$. Furthermore, after annealing, this device achieved a maximum current density of 0.49 mA/mm^2^ at 80 °C when a voltage of 1 V was applied. In the case of the annealed device exposed to accelerated illumination conditions, an ohmic regime and a maximum current density of 0.34 mA/mm^2^ under blue light were obtained. As in the ohmic regime, the current density of the device depends on the product of the free carrier density and the electron mobility. Using Equation (7), it was determined that the free electron density $n_{0}$ of the device is on the order of magnitude of $10^{18}$ $\frac{1}{{cm}^{3}}$. An activation energy of 0.12 eV for the unannealed device and 0.28 eV for the annealed device was obtained, while a resulting thermal, electrical bandgap of 2.29 eV for the device without treatment was obtained. All the previous indicates the feasibility of the use of these heterojunction device structures for flexible photoconductive applications.

## Figures and Tables

**Table 1:** Roughness, mechanical parameters, and thickness of devices.

Device	Root Mean Square Roughness, RMS (nm)	Average Roughness, Ra (nm)	Mechanical Resistance, *σ* (MPa)	Deformation, *ε*	Knoop Hardness HK
Unannealed	516.3	437.1	8.47	0.79	0.707
Annealing	686.1	594.9	5.72	0.95	8.061

**Table 2:** Ohmic behavior approximation of the unannealed device.

Light	Test 1		Test 2	
	$a_{1}$	RMSE	$a_{1}$	RMSE
Natural	0.08705979	0.00617437	0.09471705	0.00212908
White	0.04529801	0.0019811	0.04627688	0.00049613
Blue	0.03446765	0.00388346	0.02908861	0.00053178
Green	0.11644374	0.00676704	0.06317508	0.00144987
Yellow	0.16776354	0.00712602	0.12015676	0.00120055
Orange	0.16147168	0.00611561	0.14180718	0.00168812
Red	0.18024985	0.0060544	0.14394332	0.00165194
UV	0.17918761	0.00560352	0.16777995	0.00106624
Darkness	0.1780607	0.00632636	0.16046157	0.00127097

**Table 3:** SCLC behavior approximation of the annealed device.

Light	Test 1		Test 2	
	$a_{2}$	RMSE	$a_{2}$	RMSE
Natural	0.17147882	0.03089169	0.01467322	0.00023808
White	0.00976286	0.00161872	0.01311807	0.00023409
Blue	0.0254416	0.00835951	0.04990492	0.00071396
Green	0.02858158	0.00168213	0.05118732	0.00062774
Yellow	0.03048879	0.00105127	0.0334294	0.00047129
Orange	0.02773862	0.00140013	0.03134337	0.00045289
Red	0.02720028	0.00134271	0.03661899	0.00048585
UV	0.02614121	0.001166	0.03263465	0.00051688
Darkness	0.02554637	0.00071586	0.02990165	0.00048967

**Table 4:** Ohmic behavior approximation of the annealed device subjected to accelerated illumination conditions.

Light	Test 1		Test 2	
	$a_{1}$	RMSE	$a_{1}$	RMSE
Natural	0.25940246	0.01071593	0.08966493	0.00313641
White	0.33061948	0.00621719	0.25260032	0.00263576
Blue	0.32881991	0.00647173	0.2300788	0.0028575
Green	0.29996569	0.00633598	0.19416457	0.00396543
Yellow	0.25940246	0.01071593	0.1492419	0.00348888
Orange	0.25940246	0.01071593	0.13641547	0.00331512
Red	0.25180822	0.00934293	0.12487324	0.00312465
UV	0.19141202	0.02708307	0.13176643	0.00334344
Darkness	0.19261198	0.02658968	0.11371691	0.00290263

**Table 5:** Electron mobility approximation.

Light	$\mu_{n}(\frac{mm^{2}}{Vs})$
Natural	0.0210
White	0.0019
Blue	0.0071
Green	0.0073
Yellow	0.0048
Orange	0.0045
Red	0.0052
UV	0.0046
Darkness	0.0042

**Table 6:** Free carrier density approximation.

Light	$n_{0}$ $\;(\frac{1}{mm^{3}})$	
	Unannealed Device	Accelerated Illumination Conditions
Natural	5.4507 $\times \;10^{15}$	5.1600 $\times \;10^{15}$
White	2.9788 $\times \;10^{15}$	1.6260 $\times \;10^{16}$
Blue	4.9218 $\times \;10^{14}$	3.8930 $\times \;10^{15}$
Green	1.0422 $\times \;10^{15}$	3.2030 $\times \;10^{15}$
Yellow	3.0351 $\times \;10^{15}$	3.7697 $\times \;10^{15}$
Orange	3.8203 $\times \;10^{15}$	3.6751 $\times \;10^{15}$
Red	3.3192 $\times \;10^{15}$	2.8795 $\times \;10^{15}$
UV	4.3412 $\times \;10^{15}$	3.4094 $\times \;10^{15}$
Darkness	4.5313 $\times \;10^{15}$	3.2113 $\times \;10^{15}$

## Data Availability

Data are contained within the article.
